# Supplementary figures and images for: An evolutionarily conserved pathway mediated by neuroparsin-A regulates reproductive plasticity in ants
Source: PLoS Biol. 2024 Aug 12;22(8):e3002763. doi: 10.1371/journal.pbio.3002763 (PMC11398701; doi:10.1371/journal.pbio.3002763)

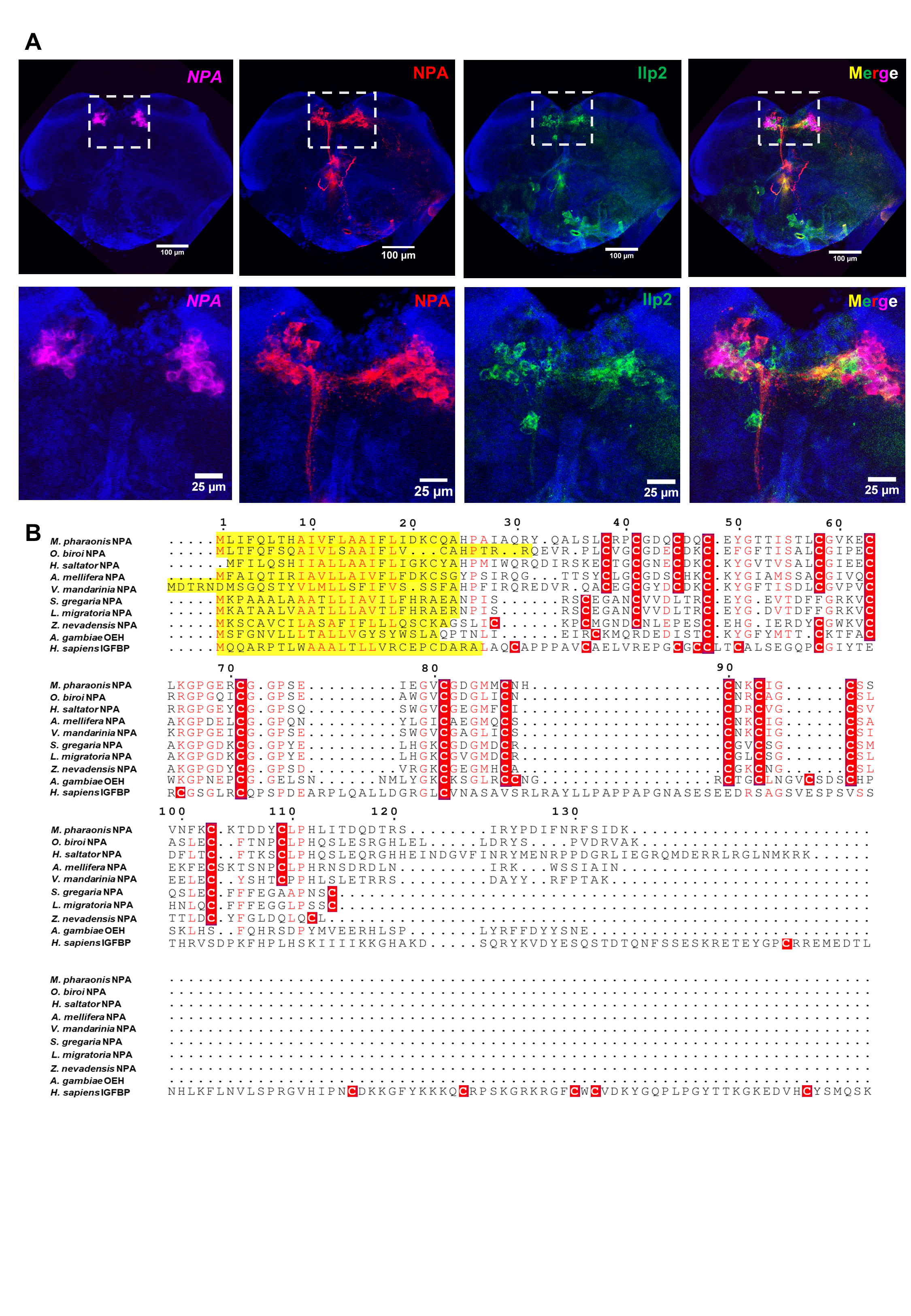

Supplement: S1 Fig — (A) HCR staining and immunofluorescence shown NPA mRNA and peptide localization in the medial neurosecretory cells (MNSC). Immunofluorescence of Ilp2 shown its co-expression with NPA. Magenta represents NPA mRNA, red NPA peptide, green Ilp2 peptide, and blue DAPI. (B) Sequences aligned by clustal W, performed in MEGA11, and decorated by ESPript 3. Conserved cysteines were indicated in white bold font and highlighted in red. Signal peptides were highlighted in yellow. Ant, bee, wasp, and termite NPA contain 14 cystine residuals, locust NPA and mosquito OEH had 12 cystines, human IGFBP had 16 conserved cystines. These sequences were downloaded from NCBI with the following accession numbers. M. pharaonis: XP_012538010; O. biroi: XP_011333498; H. saltator: XP_025154127; A. mellifera: XP_026296327.1; V. mandarinia: XP_035744017.1; S. gregaria: CAC38869.1; L. migratoria: CAA76829.1; Z. nevadensis: KDR17790.1; A. gambiae: XP_311039.2; H. sapiens: KAI4013757.1. (TIF) [file pbio.3002763.s001.tif]

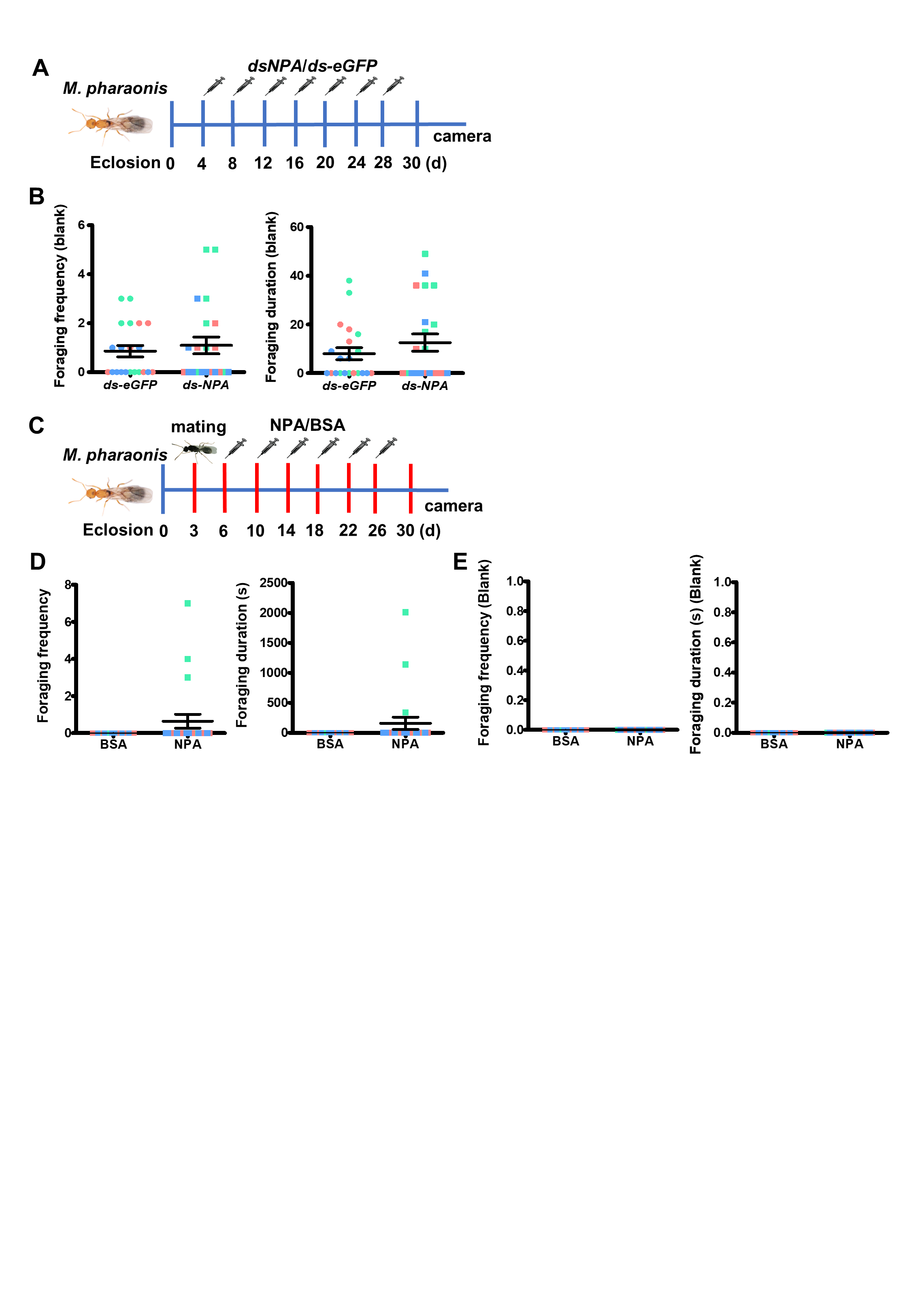

Supplement: S2 Fig — (A) The schematic diagram for behavioral assay after dsRNA injection. (B) Foraging analysis in blank area after multiple dsRNA injection as control (6–9 individuals were assayed in 1 replicate, 3 replicates, which were labeled by different colors. Each dot represents an ant, n = 21 for ds-eGFP group and n = 22 for ds-NPA group, Wald test after fitting generalized linear model, no significance). (C) The schematic diagram for behavioral assay after peptide injection. (D) Scatter dot plots shown foraging frequency and foraging duration for NPA-injected and BSA-injected groups (6–9 individuals were assayed in 1 replicate, 3 replicates, which were labeled by different colors. Each dot represents an ant, n = 20 for BSA-injected group and n = 22 for NPA peptide-injected group). (E) Foraging frequency and duration analysis in blank area after multiple peptide injection as control (6–9 individuals were assayed in 1 replicate, 3 replicates, which were labeled by different colors. Each dot represents an ant, n = 20 for BSA-injected group and n = 22 for NPA peptide-injected group). The numerical data for this figure can be found in S2 Data. (TIF) [file pbio.3002763.s002.tif]

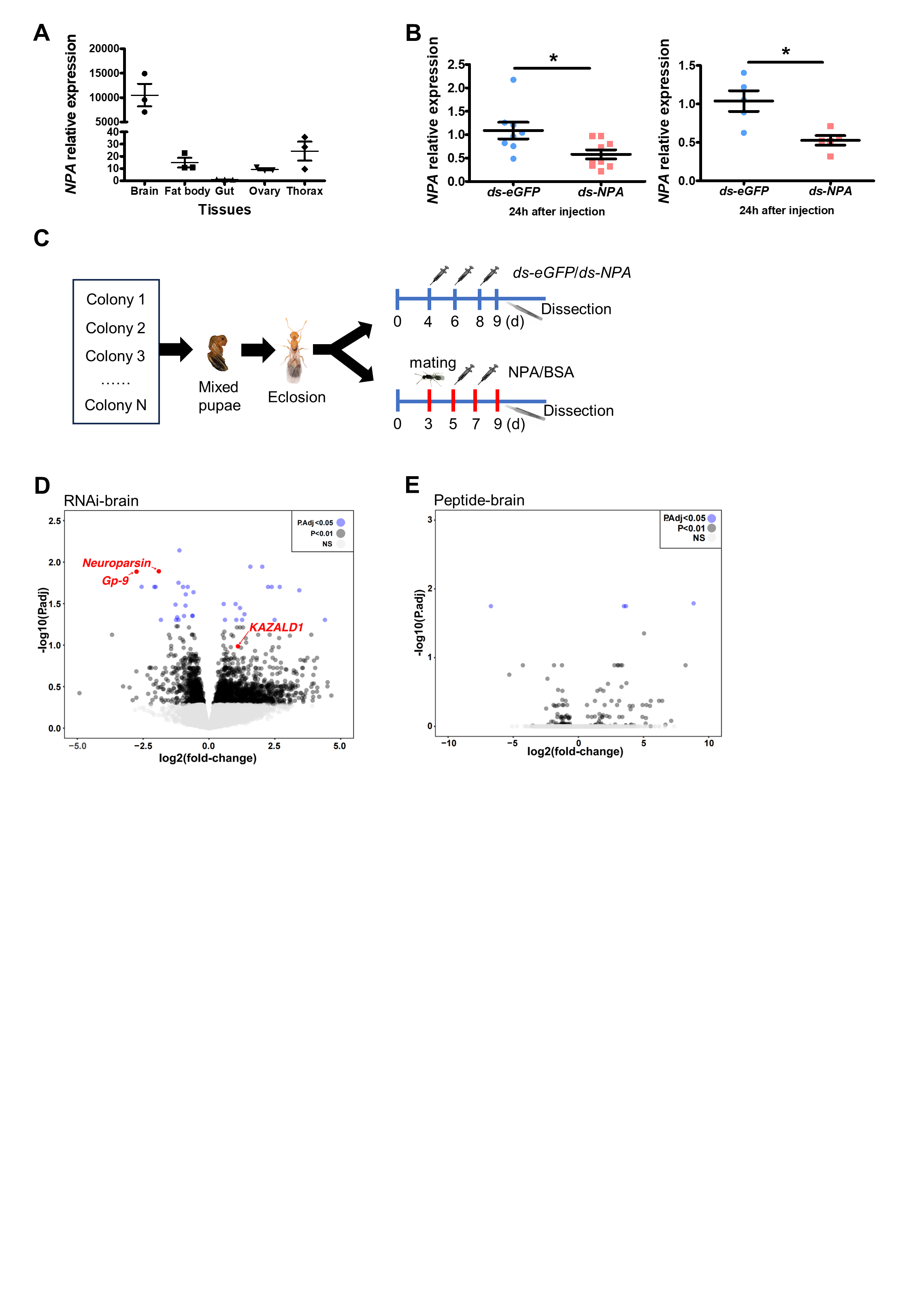

Supplement: S3 Fig — (A) The tissue-specific expression of NPA in M. pharaonis quantified by qPCR. (B) RNAi efficiency of NPA 24h after dsRNA injection, the manipulation repeated 2 times. (C) Schematic diagram for RNA-seq sample collection. (D) Volcano plot of transcriptome of gyne brains. The gynes were injected with dsRNA every 2 days, 3 times. Genes with adjusted P < 0.05 are highlighted in blue. Data are from 5 biological replicates (individual ants) per treatment. (E) Volcano plot of transcriptome of queen brains. The queens were mated on day 3 and injected NPA or BSA on days 5 and 7. Genes with adjusted P < 0.05 are highlighted in blue. Data analysis from 7 NPA-injected and 10 control brains. The numerical data for this figure can be found in S2 Data. Transcriptome data for this figure can be found in S3 and S4 Data. (TIF) [file pbio.3002763.s003.tif]

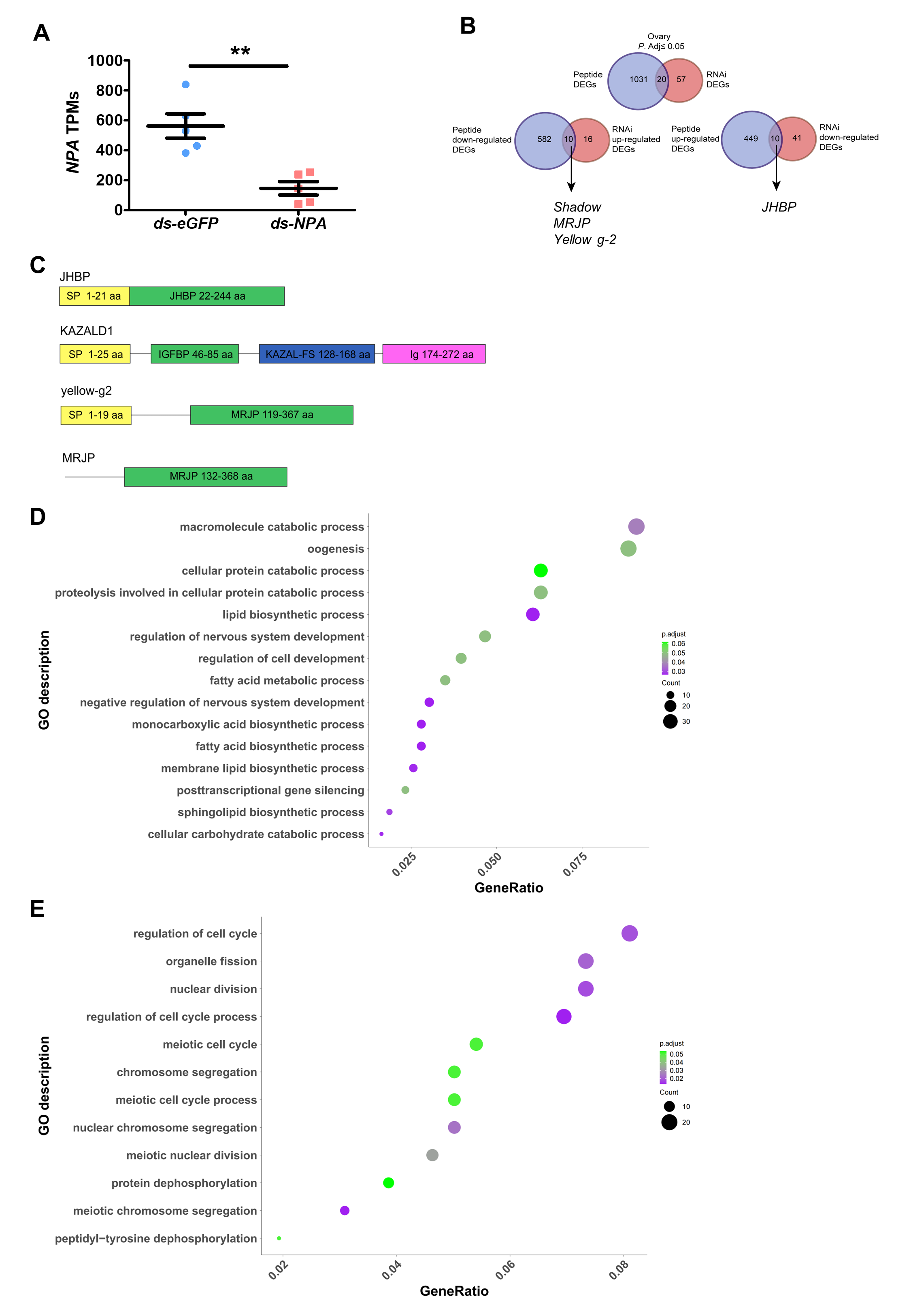

Supplement: S4 Fig — (A) NPA transcripts per million (TPMs) in the brains after multiple dsRNA injections (ds-NPA sequence were removed from transcriptomics analysis). (B) Analysis of ovary DEGs with dsRNA and peptide injections; 20 DEGs are shared between dsRNA-injected and peptide-injected groups. Among the shared 20 DEGs, 10 DEGs are up-regulated in ds-NPA-injected group while down-regulated in NPA peptide-injected group, including shadow, MRJP, and yellow-g2. Another 10 DEGs are down-regulated in ds-NPA-injected group while up-regulated in NPA peptide-injected group, including JHBP. (C) Predicted domain structures of JHBP, KAZALD1, yellow-g2, and MRJP, SP indicates signal peptide. (D) GO enrichment analysis of down-regulated genes in peptide-injected ovaries. (E) GO enrichment analysis of up-regulated genes in peptide-injected ovaries. The numerical and GO data for this figure can be found in S2 and S7 Data, respectively. (TIF) [file pbio.3002763.s004.tif]

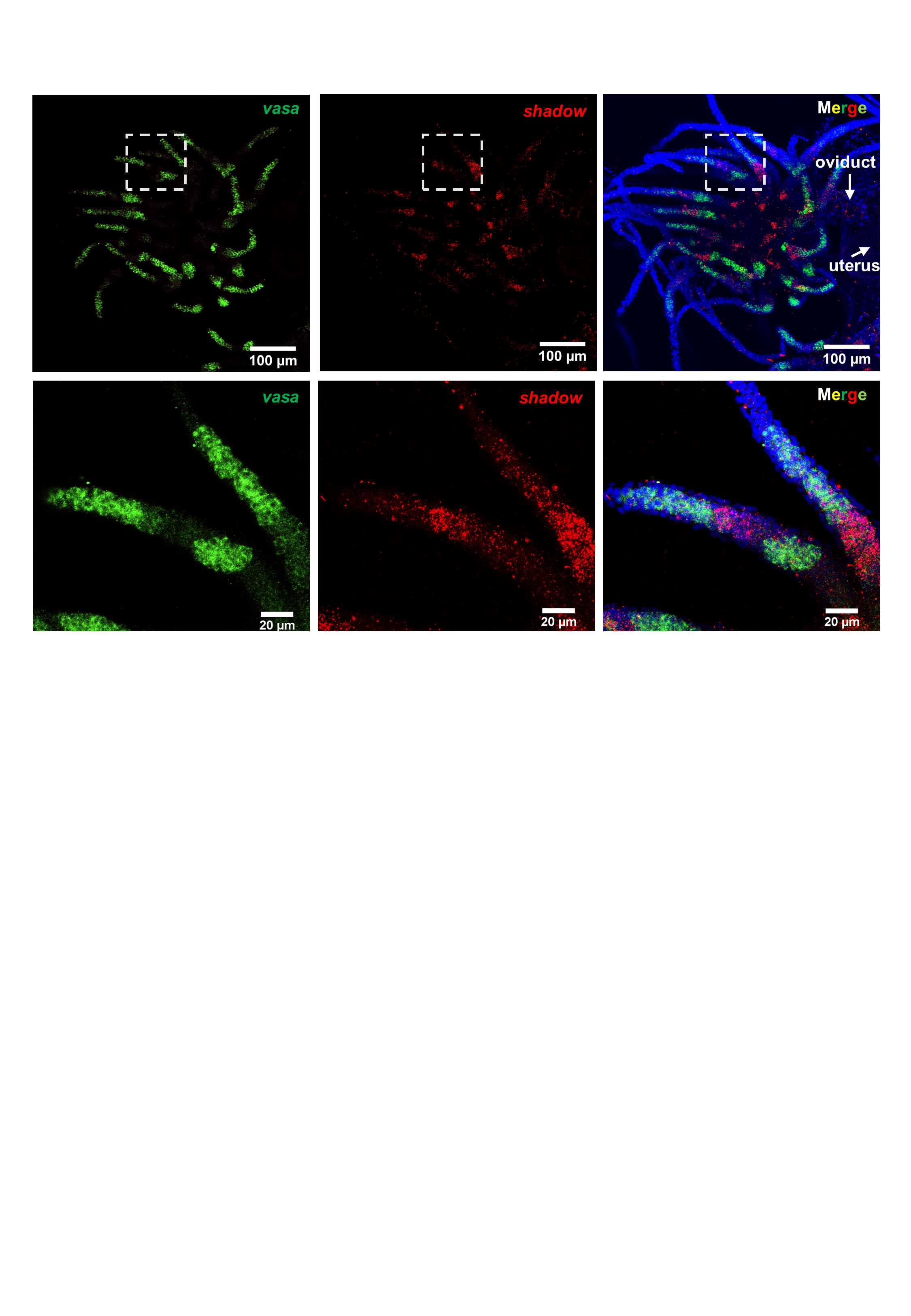

Supplement: S5 Fig — Shadow and vasa were primarily expressed in distinct segments in the ovarioles. Green, red, and blue represent vasa, shadow, and DAPI, respectively. (TIF) [file pbio.3002763.s005.tif]
