# Supplementary material for: An evolutionarily conserved pathway mediated by neuroparsin-A regulates reproductive plasticity in ants
Source: PLoS Biol. 2024 Aug 12;22(8):e3002763. doi: 10.1371/journal.pbio.3002763 (PMC11398701; doi:10.1371/journal.pbio.3002763)
Supplement: Attachment — Submitted filename: Response to reviewers.pdf [file pbio.3002763.s020.pdf]

REVIEWERS' COMMENTS:

Reviewer #1:

In this manuscript, the authors claim to uncover the role of a single gene/peptide, Neuroparsin A (NPA), in controlling "reproductive-role differentiation in ants". The authors show that *NPA* expression differs between reproductively active and virgin queens in several species of ants, and then perform NPA manipulations that cause changes in reproduction and behavior, all aligned with the queen function. While I am convinced that the authors do show that NPA regulates reproduction but I am not convinced by their claims that it also regulates behavior.

Response: We thank the reviewer for all these constructive comments which help to improve our revision.

The authors report a series of experiments that reveal the anti-gonadotropic function of NPA. These are well-executed manipulations that provide experimental evidence that NPA inhibits reproduction. It is also interesting to note that NPA may be colocalized with ILP2, which was recently shown to activate reproduction. However, the second pillar of the manuscript, namely that NPA regulates behavior as well, is not well supported by the data. The behavioral experiments have several flaws that prevent from making firm conclusions on the behavioral role of NPA. First, all behavioral results may be associated with reproductive changes (e.g., locomotory activity being reduced in reproductive individuals) and would simply be the indirect consequence of the effect of NPA on reproduction.

Response: Thanks for the reviewer's comment. In our manuscript, we have not excluded the possibility that NPA may indirectly affect behaviors, actually, we propose that the brain-ovary axis underlying reproductive regulation may implicate both forward and backward signalings. We speculate that the signalings from the ovary (or fat body) may feedback to regulate brain functions, so that behavioral outputs of the ants would be affected. There is growing evidence in insects as well as mammals that female reproductive states could affect their behavioral outputs through the actions of circulating hormones in modulating the neural circuits of the brains [1-5]. The reproductive ground-plan hypothesis proposes that the co-option of the regulatory components for female reproduction may underlie social evolution [6, 7], and the central prediction of the hypothesis is that the reproductive physiology and social behavior, in particular division of labor, are linked by pleiotropic genetic network [8, 9]. In agreement with this hypothesis, our study demonstrated that this conserved pathway mediated by NPA has been used to regulate the division of labor on both reproduction and social tasks between castes in ants. Consistently, previous study in *Harpegnathos saltator* ants also demonstrated that another brain peptide corazonin negatively regulates *vitellogenin* expression in the fat body of the workers, and the altered *vitellogenin* signaling could affect reproduction as well as hunting behaviors of worker ants [10]. While we agree with the reviewer that further study on whether the behavioral effects of these neuropeptides are direct or indirect is an interesting question to follow up, such question could only be addressed with advanced genetic tools for tissue specific manipulations (e.g. restricting the functions of NPA within the brain, or alternatively, specifically disrupting the signalings from the ovary or fat body), which is currently non-developed yet in ant systems. We re-wrote the introduction to highlight the potential role of brain-ovary axis in regulating developmental plasticity in ants.

Second, because of the way they are scored, all behavioral measurements (activity, foraging and brood care) may simply reflect variation in locomotion (more active individuals are more likely to interact with food or brood). Proper behavioral investigations would control for such variation.
Response: We scored foraging behavior as more than 3 seconds contacting with the food and brood care behavior as more than 3 seconds touching the brood or pupae. To address this question, we applied the same measurements to score the contact frequencies of the ants to the similar-sized blank areas near the food patch as controls. If the high foraging is simply due to the locomotion, we would expect the high locomotion group would also have high frequent contact in the control region. We found that the ants display comparable contact frequencies to the blank areas, indicating that the higher foraging of *ds-eGFP* ants are not directly caused by their higher locomotion activities. The new results are appended to the S4 Figure. Another evidence is that, the NPA peptide-injected ants display significantly higher locomotion activities (Fig. 3F), however, they rarely display any foraging activities (S3 Fig.). If the foraging activities directly reflect locomotion differences, then we should obtain similar measurements for this scenario, however, our actual observation indicates the speculation is not true.

Third, all behavioral experiments are performed one after the other using the same individuals in the same box, thus variation in one behavior may directly trigger variation in the next one that was tested (e.g., higher foraging activity could result in better nutritional status or larger food reserves to be shared, resulting in apparent increase in brood care).
Again, proper behavioral investigations would separate the different experiments to disentangle the effect of the different behaviors.
Response: We performed the behavior assays one after the other with the same cohorts of ants primarily due to the technical challenges and limited gyne samples for experiment. Collecting enough simultaneously eclosed gynes requires synchronized set up of hundreds of ant colonies, which would take almost one year, since the gynes are only occasionally reared by the workers in the absence of the queens. Besides, multiple injections must be performed to induce long-term *NPA* knockdown, which will cause notable losses of the gynes during the manipulations. Considering these difficulties to address this question, we removed the brood contact behavior data in the current revision. This change does not affect our conclusion on other behaviors.

Finally, multiple ants were put together in the same box for behavioral essays, resulting in dependency of the data, which is very problematic in social animals (the behavior of one individual would be greatly influenced by the behavior of the rest of the group). In my opinion, the behavioral experiments are very weak in comparison to the molecular work and are not on par with the usual standards of Plos Biology.
Response: Social insects live their entire life centered around colony activities and they must constantly interact with other nestmates. The reason we performed the behavioral experiments with groups of ants is because isolation is an extremely stressful environment for social insects. As observed in our study as well as reported by other research groups, isolation could cause pronounced behavioral abnormalities, as well as gene expression fluctuations in social insects, and their health states are impaired [11-13], therefore, we have to perform the behavior

experiment under the natural condition as a social group to avoid the impact of the unnatural manipulation by isolation. And since we measured the behavior scores for all individuals in the knockdown groups and the control groups, the significant difference results between the two groups should be sufficient to reflect the behavior effects of the gene knockdown.

Apart from this main concern, I have other concerns that I detail below.
L2-3: I think that the title is not very clear, and could be improved. Furthermore, there is a typo in neuroparsin…
Response: Thank you for the suggestion and careful reading. We have corrected the spell of neuroparsin.

Generally, I think that the writing could be improved substantially. The manuscript was very difficult to read and understand in some parts.
Response: Sorry for the confusion, we carefully polished our writing.

L40-59: I find the use of terms such as "queen-like" or "worker-like" in the abstract very vague, and not appropriate for a generalist journal like Plos Biology.
Response: We have rephrased the sentence to "Knock-down of *NPA* in gyne enhances ovary activity and inhibits activity for locomotion and foraging behavior. Conversely, injection of NPA peptide in fertilized queen suppresses ovary activity and causes higher locomotion.". (L46-48)

L40-59: I do not think that the claim "we found the function of NPA in regulating reproductive role differentiation is conserved across the ant phylogenetic tree" is supported by the data. The number of species studied is too low and the species list is not representative of the ant phylogeny (e.g., two Camponotus species are used to represent the entire subfamily Formicinae).
Response: We do acknowledge that the number of species we studied was limited compared to the huge diversity of ants. Since the gyne individuals of ants only appear in very short periods in the wild, it is rare that we can collect the gyne samples from many species for experiments. However, to satisfy this reviewer, we have added other two species *Oecophylla smargdina* and *Lasius alienus* from subfamily Formicinae for rt-qPCR analysis. The new results are included in the Figure 1. We found that the *NPA* expression levels were also significantly higher in gyne than queen in *Oecophylla smargdina* and *Lasius alienus*rom. All together, the currently available species have covered 5 subfamilies and 8 genus.

L100-103: From the introduction, it sounds like NPA has already been identified as a candidate for reproductive division of labor in previous studies, which appears to be redundant with some of the results presented in this manuscript. I think that the only added value of the manuscript is the experimental evidence that NPA regulates reproduction. While it is very interesting for ant researchers, I am not convinced that this finding will elicit a broad interest.
Response: We have edited the introduction to specify what is known about *NPA* and what questions have not been addressed. Previous studies only demonstrated the potential correlative relationship between *NPA* expression levels and reproductive levels. Whether and how NPA is

causally responsible for reproductive role differentiation, and whether the functions are conserved across different ant species with distinct reproductive differentiation scenarios remain to be clarified. In this study, we expanded the number of species to cover five different subfamilies and eight genus, and provided experimental evidences to reveal the causal role of NPA in regulating reproductive physiologies as well as behavioral performances. Our study suggested that NPA functioned as a crucial component through the brain-ovary axis for the reproductive role differentiation, and demonstrated this function was conserved across ant species that have distinct reproductive role differentiation scenarios. As NPA is a conserved IGFBP (IGF binding protein) across diversified arthropods, the findings of our study demonstrating the function of NPA as a neuro-hormone integrating juvenile hormone and ecdysone pathways would be of interest to a wide range of researchers in insect physiology as well as to a general readership.

L129-138: Similar to my previous comment, it is not clear what is novel vs published data in this part of the results.
Response: These sentences in the introduction show three models of reproductive role differentiation in adults ants proposed in Nagel et. al [14]. In these three models, a correlative relationship between *NPA* expression and reproductive activation was reported in three species. In this study, we surveyed an expanded list of ant species covering 5 subfamilies and 8 genus, and demonstrated that the correlative relationship between the *NPA* expression and reproductive activation is conserved across all these ant lineages. More importantly, we provided experimental evidence to show the functional roles of NPA in both physiological as well as behavioral changes associated with reproductive role differentiation.

Figures: Maybe it is due to the pdf conversion, but the figures are of very bad quality, to the point where it is difficult to decipher the labels and assess the results.
Response: We are sorry for the low-quality of the figures that made the reading difficult. The issue was caused by the improper conversion from a word to a pdf document. We fixed the issue with improved figure quality in the new version of the manuscript.

Figure 1: The evidence for differential expression of NPA is actually weak in many of the species. In particular, in Monomorium pharaonis - the main study system - it looks like the effect is entirely driven by a single data point. This should be clearly acknowledged. I am not convinced that the authors can make a general, robust claim of a central regulator of role differentiation when there is very weak evidence that it actually differs between gynes and queens.
Response: We have repeated this experiment with *Monomorium pharaonis*, and the result was consistent with the previous one. We have updated the result in Figure 1. The weak differential expression is mainly due to the small sample size, which was mainly caused by the difficulty in sample collection, since most of the ant species were collected from the fields and the gyne/queen samples were very limited in the colonies.

Figure 1: I do not understand why reproductive model 3 is in this manuscript. It seems out of context because it is outside the scope of the main point of the introduction: that insemination

triggers reproduction and behavioral changes.
Response: The model 3 represents some of the secondarily derived ant lineages such as clonal raider ant *Ooceraea biroi* where their workers regained reproductive ability without insemination. *Ooceraea biroi* reproduce by thelytokous parthenogenesis, alternating between an egg-laying reproductive phase to a brood-caring non-reproductive phase. In this species, the components of GRN for reproductive role differentiation that is normally not expressed in the non-reproductive phenotypes might have been co-opted for their respective worker reproductive role transitions. Although in this model, the transition is triggered by the presence/absence of the larval signals rather than the insemination. Although we did not perform a functional experiment on this species， our own expression data also supported the *NPA* expression is also correlated with the reproductive status in this species, which might also through this conserved brain-ovary axis GRN.

L141: "p value" is mentioned twice. Why?
Response: Sorry for the confusion. Actually, we described the results of four different species here. To clear the vagueness, we deleted the descriptions of *p*-values in the main text and appended the exact corrected p-values in the supplementary notes.

More generally, throughout the manuscript, the authors should report the effect size and the exact corrected p-value, not just "P<0.05". This does not follow standard scientific practice and is misleading, especially as the evidence here is very weak.
Response: We have appended the exact corrected *p*-values, the Cohen's d-values for effect sizes, and 95% CI to the S5 Data.

L153: I am not convinced by the general use of "reproductive activated role" in this manuscript. This is not a well-accepted term in the literature and it does not have an established meaning. The authors could simply state that it correlates with reproduction (especially as they provide very weak support for a behavioral role, see before). Or that it differs between reproductive and non-reproductive individuals. I understand that they want to avoid the confusion with worker vs queen comparison but I think they make too much out of a simple comparison.
Response: Thanks for the suggestion, we have changed “reproductive activated role” simply to the “reproductive activation in ants”. (L149-150)

Maybe I missed it, but I did not find proper evidence for all the experiments that the experimental manipulations did result in a downregulation of NPA expression or increase in the NPA peptide quantity.
Response: The *NPA* expression levels in knockdown manipulation were added in the S5 Figure. The NPA peptide level has not been quantified, since the endogenous NPA peptide and injected NPA peptide were too low to be detected by western blot. We have added another control with heated inactive NPA for the reproduction experiment in *Monomorium pharaonis*, which showed no effect in suppressing reproduction. The ovarian activities of the queen injected with heat-inactivated NPA were similar to those injected with BSA, indicating that the reproduction suppression was caused by active NPA effects.

Finally, coming back to my main concern, I cannot help but notice that the analyses of the transcriptomic response to the experimental manipulation and the discussion in general (including Figure 6) focus almost exclusively on the reproductive role of NPA. To me this study is about the impact of that gene on reproduction, not on a complex behavioral repertoire associated with reproductive role differentiation.
Response: Thanks for the reviewer's comments. These constructive comments greatly help to improve our rationale of the research design, as well as the presentation of our findings. We have extended our responses to these major concerns in previous paragraphs. Please refer to our point-to-point responses to these major concerns in the beginning paragraphs.

Reviewer #2:
This manuscript builds on previous work (Nagel et al. 2020) that implicated a gene encoding neuroparsin A (NPA), among others, in association with early regulation of reproductive activation in adult female Monomorium ants. The prior results were largely correlational, but the NPA correlation was established on a fine spatiotemporal scale in the brain. The current study expands upon the prior results by showing that the association between reproductive activation and NPA decrease is persistent across 5 subfamilies of ants encompassing three distinct modes of reproductive activation (queen/post-mating, gamergate/dueling, clonal/cyclical), and interrogates the function of NPA with a series of experiments involving NPA knockdown and NPA peptide injection in i) an ant from the myrmecine subfamily with a "typical" ant reproductive life cycle and ii) an ant from the ponerine subfamily with dominance-based reproductive gamergates. The results reveal that NPA suppresses reproduction by being involved in signaling from the brain to the ovaries and regulates behavior shifts in the myrmecine ant M. pharaonis and the ponerine ant H. venator, consistent with a model in which NPA has a conserved function in regulating reproductive role differentiation across ant species. The work is impressive in scope and design. The authors have leveraged phylogenetic variation in ant life histories to reveal an apparent conserved molecular underpinning of ovarian activation and affiliated behaviors that may be highly important to the temporal regulation of reproduction and behavior, and thus division of labor, across ants. I would definitely suggest this paper as a reading for any lab group working on ant biology, social insects, or temporal regulation of reproduction and behavior in animals (especially in insects). The experiments that involve multiple rounds of treatment with RNAi and peptides are particularly impressive and are not something most well-funded research groups could pull off. The work required extensive expertise in animal husbandry, behavioral experimentation, ant injection/dissection, molecular genetics, and bioinformatics. The results were clearly presented in figures and clearly described in text. I commend the researchers and very much enjoyed carefully reading this important research. I think it is suitable for PLOS Biology.
Response: Thanks for the encouraging comments. We are very appreciative of these positive statements.

I did not review the supplementary files; they were in a .rar archive and I would have had to download a new app on my laptop to decompress. I did not evaluate the manuscript for

grammar.

Reviewer #3:
This study provides an interesting and overall straightforward exploration of the effects of NPA on ant behavioral plasticity. Strengths of the study include initially considering 7 ant species across the ant phylogeny and the relatively simple and straightforward experimental design. Unfortunately, the figure quality in the pdf I had access to for review was relatively very low so that I couldn't make out axes labels and figure details for all of the figures. That said, the results appear to support the conclusions. I did not find any major problems with the study design but had some detailed questions (e.g., about mating status of gynes). My main comments / suggestions for improvement are regarding several statements that are incorrect or overstatements throughout the manuscript. Details below:
Response: We are sorry for the low-quality of the figures, which was caused by improper conversion of a word to a pdf document. We have fixed the issue and the quality of the figures is improved in the new version of the manuscript.

Figure 1 indicates that all red species have workers with "ovary lost". This is not correct.
Response: Thanks for your careful reading. We realized “ovary lost” could not generally be applied to all the species labeled in red, we have deleted “ovary lost” in the new Figure 1.

Several statements in the Introduction that provide background / motivation are unsupported or incorrect:
L68-70 and the Abstract states that reproductive caste differentiation and "reproductive role transition" are different. The latter is described as "phenotypic plasticity" L70 and Abstract, while the former is described as L43-44 ". realized by canalized development". Both are clear examples of phenotypic plasticity.
Response: Thank you for this comment. Reproductive caste differentiation is a bifurcation developmental process under canalization that results in permanent and irreversible morphologically differentiated castes. While reproductive role differentiation is induced by insemination or other social signals in adulthood which entail physiological and behavioral plastic changes. In this manuscript, we have emphasized the conceptual differences regarding reproductive caste differentiation and reproductive role differentiation, since these two concepts are easily confused to a general readership. Reproductive caste differentiation primarily refers to the differentiation between gyne and worker, which is an irreversible consequence of development. Reproductive role differentiation happens in adulthood, after the morphological caste has already been determined. We have discussed this difference in our previous study [14]. In our revised manuscript, we have rephrased the relevant descriptions and made it clear that the reproductive role differentiation is an example of phenotypic plasticity which entails physiological and behavioral plastic changes in adulthood.

L86-L88 this is an overstatement. The GRN underlying gyne/queen differentiation is largely not understood and there is only some evidence for a conserved GRN across ants.

Response: Thanks for the comment. We have toned down our statement and rewritten this sentence, and it now reads “Previous comparative transcriptomics analysis has identified a repertoire of differentially expressed genes (DEGs) underlying gyne/queen transition, and most of these genes display similar directional expressions in different reproductive roles in the distantly related ant lineages with reproductive workers, suggesting the co-option of the same gene regulatory network (GRN) to regulate different forms of reproductive role differentiations across ant species”. (L93-98)

L132-L138 please provide references for to support these statements

Response: We have added references for the three represented reproductive models. The study by Nagel et. al [14] has used *Harpegnathos saltator* to represent the worker/gamergate transition model and *Ooceraea biroi* to represent the non-reproductive/reproductive worker transition model. The study by Heinze and Tsuji has reported that the genus of *Harpegnathos* and *Gnamptogenys* species have gamergates[15]. In the book chapter by John Lattke [16], the author provided the evidence to support reproduction through gamergate in *Gnamptogenys bicolor*.

L139-144 this statement requires support. In contrast to this claim, an unpublished ms on biorxiv (Meurville et al 2022 https://www.biorxiv.org/content/10.1101/2022.09.25.509371v1) actually concludes that the ancestral state for ants is the presence of gamergates (mated workers).

Response: We have made this statement primarily referring to the report by Peeters and Schmidt (Multiple evolution of gamergates (sexually reproducing workers) in the ant subfamily Ponerinae, Conference: XVI Congress of the International Union for the Study of Social Insects, CopenhagenAt: Copenhagen, DenmarkVolume: p. 96, 2010), in which the authors had claimed that “A detailed molecular phylogeny of the Ponerinae revealed that species with gamergates occur in 14 “genera”, including seven unrelated lineages of the non-monophyletic genus Pachycondyla. These taxa are broadly scattered and are nested within clades of AQ (alate queens monopolize sexual reproduction) species. Reconstructions of ancestral states indicate that gamergates evolved independently a minimum of seven times within Ponerinae, and probably many more. These analyses strongly suggest that the MRCA (Most Recent Common Ancestor) of extant ants lacked gamergates (BPP = 0.998), and that the MRCA of the ponerine tribe Ponerini (containing 13 genera with gamergates) also lacked gamergates (BPP = 0.962). Hence gamergates are derived both within ants generally, and ponerines (as well as ectatommines) specifically”. We have also read the new manuscript by Meurville et. al, in which the authors had performed ancestral state reconstruction with 416 ant species to interrogate the presence (or absence) of reproductive workers in the common ancestor of all ants. The authors claimed that “gamergates are not secondarily evolved, but rather that reproductive workers were present in the common ancestor to all ants”. However, we also notice this manuscript has not been formally published yet. Considering this is still under debate, we removed the word “derived” anywhere relevant.

L142, provide citations that "Worker/gamergate transition is a derived model"

Response: Please refer to our answers to the previous point. We realized that whether the

worker/gamergate reproductive model is secondarily evolved is still under debate. Thus, we deleted the word “derived” anywhere relevant.

L187 "We collected newly-eclosed gynes…". Were these gynes in colonies that also had males? If so, how do you know that they were not mated?
Response: We separated old pupae of the gynes from the colonies, and collected newly-eclosed gynes every day. This method ensures that our experimental gynes were not mated.

L201 please clarify what "over-numbered males" means. I think this means that you added more males than gynes, but please provide more details about exactly how many of each were used.
Response: The ratios of males to gynes were around 1.5:1. To clear the vagueness, we added annotation to the “over-numbered” as “over-numbered (gyne:male≈1:1.5)” in the new manuscript (L195-197). For each experimental and control group, we collected ~60 newly-eclosed gynes and ~90 males on day 0, we let them mate on day 3, remove the males on day 4, then inject the peptide on day 5 and 7. We excluded the individuals that are not successfully inseminated from our analysis (by checking the spermatheca). We have added the detailed description in the methods section.

After gynes and males were together, how did you confirm that the gynes had mated? Is it possible that some of the gynes were actually unmated?
Response: We have dissected the reproductive system and checked the status of the spermatheca to confirm whether the female was indeed inseminated or not. Unmated gynes had empty spermatheca, so that they were excluded from the analysis for queen reproductive and behavior phenotypes (In the behavioral assay, we labeled different individuals with different color codes, so that we could identify each individual when examining its spermatheca status after the assay).

L241-L258 these knockdown studies are in *M. pharaonis*, correct? Please clarify this in the text.
Response: Thanks for the suggestion. We have clarified these statements, and it now reads “To address whether consistently low *NPA* level is necessary for stabilizing queen behaviors, we conducted long-term *NPA* knockdown in *M. pharaonis* by injecting *ds-NPA* into hemolymph of gynes every four days for seven times in total and injected *ds-eGFP* following the same procedure as control group, until the gynes become 30 days old”. (L239-242)

L329 "...in derived ant lineages such as H. venator". What is a derived ant lineage?
Response: We realized the statement on the presence or absence of reproductive worker in the common ancestor of ants is still under debate. The same concern was raised on former paragraph. We have deleted the word “derived” wherever relevant.

L350-L370 please provide details for biological / technical replicates
Response: Thanks for this suggestion. We have added details on the biological replicates for transcriptomics analysis, it reads “For dsRNA injection, we analyzed 5 knockdown and 5

control brains, and 7 knockdown and 6 control ovaries. For peptide injection, we analyzed 7 NPA-injected and 10 control brains, and 10 NPA-injected and 12 control ovaries”. (L340-342)

L498 please use the scientific name instead of "fire ant"
Response: Thank you for this suggestion, we have used the scientific name of *Solenopsis invicta* in the text.

L576 please clarify the "customized diet"
Response: Thanks for this suggestion. We have clarified the customized diet in the method section “60g sucrose (Sangon, A502792-0500), 6g agar (Sangon, A505255-0250) and 2 tablets of vitamins with minerals dispersible tablets 21 (Kangkang, 10051475686242) heated and dissolved in 500ml ddH_20. The liquid diet is then poured into petri-dishes to cool down, then stored at 4℃”. (L565-569)

Reviewer #4:
In this paper, the authors hone in on a neuropeptide gene, Neuropasin, which was previously identified as being differentially expressed between whole bodies of queen and worker ants, and which has been previously shown to affect reproductive development in locusts. One reference suggests that the Drosophila version of this gene is an orthodox of human insulin-binding protein.
The authors ask whether Neuropasin may be a key regulator of reproductive development in ants. They perform multiple experiments across several species, notably finding via qPCR assays that higher NPA expression correlates with greater ovarian development.
They also focus on Monomorium pharaoh ants where they artificially manipulate NPA levels by injecting NPA protein (emulating higher expression), or dsRNA (to inactivate). They find effects at several levels:
- Foraging: more foraging when NPA is injected. Less with dsRNA inactivation.
- Oocyte number: less when NPA is injected. More when dsRNA inactivation
- Oocyte size: lower when NPA is injected. More when dsRNA.
And perform similar analysis in Harpegnathos where ovaries of subordinate workers can become activated in the absence of a queen. They find similar patterns there.

These analyses are commendable, and do implicate NPA in ant biology. There have been only few good functional studies in ants, and this one uses a variety of techniques to try to disentangle what is going on with a particular gene.
Response: We appreciate the reviewer’s positive comments on our study.

However, I struggle with several aspects.
Notably, I feel the controls could be better. I keep asking myself "what if serotonin or adrenalin or a poison had been injected"? Surely we would see similar changes to what we are seeing with the injections of NPA or dsNPA. With these kinds of animals, ideal experiments are more difficult that with Drosophila or yeast. Most experiments here have a single control treatment (e.g., injection of dsGFP or of BSA). one or more additional controls (e.g., targeting other

genes that are not thought to be involved in reproduction, or injecting ineffective versions of the same gene) would create more trust. "Normal" transgenics is difficult in this system.
Response: Following the reviewer's suggestion, we have added *ds-random* (randomized sequence of *NPA*) and heat-inactivated NPA peptide as controls for RNAi and peptide injection manipulations, respectively. The new results have been appended to Figure 2. We obtained similar results with these controls. The randomized sequence of *NPA* was designed on http://www.detaibio.com/sms2/random_dna.html, which used the original *NPA* CDS as template to obtain a shuffled sequence of *NPA*. Chemically synthesized *NPA-random* sequence was ligated into a vector, which was then used as a template to generate *ds- random* by in vitro transcription.

The authors also perform tissue-specific gene expression analyses in brain and ovaries after functional manipulation. This shows some correlative effects, with key candidate genes showing changes in line with the model that the authors suggest in Fig 6. However, hundreds of other genes show similar changes, so the general pattern does not demonstrate causality of the particular interactions outlined. This feels like extreme over interpreting.
Response: We have identified 77 and 1051 DEGs expressed in the ovary after knockdown and peptide injection (padj<0.05), respectively, and only 20 DEGs were shared between these two manipulations. Among the 20 shared DEGs, 10 of them were up-regulated in dsRNA treatment while down-regulated in NPA peptide treatment, and other 10 DEGs displayed the reversed patterns (S5 Fig). The genes we selected for further investigation are from these 20 shared DEGs and have high log2 Fold change values. Besides, we also performed RNAi to knockdown one of the top candidate genes (*JHBP*), and the ovarian activities of the ants were significantly promoted, supporting the role of *JHBP* in suppressing reproduction. We have toned down the description of the proposed model in our revised manuscript. (L386-387 and L392-396)

There are issues with language that I will ignore.

Thus overall, I think this study is overreaching. To stand in Plos Biol, I feel that better controls are needed, and the interpretations need to be substantially reduced (or more targeted data may be needed - e.g., evidence of how the supposed gene regulation may actually work).
Response: Thanks for the suggestion. We have carefully polished our writing and toned down several of our statements.
Additional issues:
- L48 - why neuorparsin A and not some of the (hundreds?) of other differentially expressed genes
Response: We realized that our writing in the abstract does not flow smoothly, we carefully polished our writing in the abstract and throughout the main text. We have extended the rationale on the selection of NPA to study in the introduction section. Firstly, *NPA* is a top ranked gene in the DEGs list generated from gyne and queen transcriptomics comparisons in *Monomorim pharaonis*. Secondly, *NPA* is one of the earliest differentially expressed genes after insemination in 5 day old queens, and it displayed a long term consistent suppressed expression in the queen from day 5 to day 30. Thirdly, NPA suppression is correlated with reproduction activation as reported in previous studies with a few species. Finally, NPA

homologs in other insects have reproductive regulatory functions. Therefore, we selected NPA as our top candidate for in-depth investigation. We have extended these rationale in the revised introduction section.

- L79-80 indicates that fully-functional queens who fail to mate would usually die soon this is a strong non-intuitive statement that wants supporting references.

Response: Thanks for your suggestion. We realized that the statement caused confusion here, and what we mean here is that the gynes who failed to mate during the nuptial flights could not found the colony and usually die afterwards. To clear the vagueness, we deleted the sentence "while those who failed would usually die soon afterwards".

- L511-514 - unclear what is being said here, but it sounds like its an important speculation

Response: Thanks for your suggestion. We have rewritten this sentence. Now, it reads as "In the same sense, pharaoh ant queens display extremely stereotyped behaviors which might partially be due to the impaired pheromonal sensation caused by *Gp-9* down-regulation.". (L499-501)

- Why no Drosophila data about this gene? I guess they've lost it? Make that clearer.

Response: Thanks for this suggestion. The NPA gene was lost in several *Drosophila* species, including *Drosophila melanogaster*. We have added the description in the introduction section. (L102-104)

- L141 - what are these 2 p-values?

Response: Sorry for the confusion. Actually, we described the results of four different species here, there are four *p*-values. To clear the vagueness, we deleted the descriptions of *p*-values in the main text and appended the exact corrected *p*-values in the supplementary data.

- L140 - make it clear that is from whole bodies. Which is difficult to interpret - as ovaries in queens are bigger than genes. It could be that NPA is simply expressed during oogenesis or early development rather than differences in the brain. WE would expect this pattern for hundreds of ovary-expressed & meiosis genes.

Response: Thank you for this suggestion. We performed rt-qPCR experiments with different body parts of pharaoh ants (brain, fatbody, ovary, gut and thorax), and found that *NPA* is specifically expressed in the head (S5 Fig.). We also performed HCR and IHC staining with the brain as well as ovary of pharaoh ants, and we only detected NPA positive signals in the brain. These results indicate that *NPA* is specifically expressed in the brain, while not in the ovary of ants. For quantifying NPA expression in other ant species, we only used their heads for rt-qPCR experiments, while their other body parts were discarded. Therefore, the differentiated expression of NPA presented in the figure was not caused by ovary differences between gyne and queen, instead, it reflected their brain differences. We have clarified the brain-specific expression of NPA by re-writing the sentence as "Among the six species with gyne/queen transition model, the *NPA* expression level in the brain is always down-regulated in queens compared to gynes". (L137-138)

- L156-7 unclear if the authors are saying that NPA is exclusively expressed in the brain. Wording is misleading/ambiguous

Response: NPA was exclusively expressed in the medial neurosecretory cells in the brain. We have clarified this in revision.

- L167/Fig S1 - similarity to IGFBP is unconvincing (also no statistical measure of confidence that this is true).

Response: NPA and other parsin family members contained 12-16 conserved cysteine residuals, which also is one of the characteristics of IGFBP. Furthermore, the evidence in *Schistocerca gregaria* has proved neuroparsin could bind insulin-related peptide [17]. The method for multiple comparison of amino acid sequences refers to Veenstra's [18] analysis of neuroparsin in multiple insects. We have cited these literatures in reference.

- L285-6; Fig 3 - this was done on groups of individuals. The stats shown treats them individually. This is pseudo replication. Instead use an analysis model that incorporates 2"group" as a random factor.

Response: Thank you for this suggestion. We performed the locomotion experiments for three times. Each time, we separated individuals from the same colony into experimental group and control group. The experimental group were injected with *ds-NPA* while the control group were injected with *ds-eGFP*. After the knockdown manipulation, the locomotion of individuals were tracked. Following to the reviewer's suggestion, in this revision, we didn't combine the three experiments, instead, we presented the results from just one experiment in the main figure and presented the other two experiments in the supplementary data. All three experiments display similar results. For the peptide injection manipulations, we accordingly changed the presentations.

- citation 42 is off-topic and predates the discovery of a supergene by 10 years (!)

Response: We have replaced the reference to the following: Wang J, Wurm Y, Nipitwattanaphon M, Riba-Grognuz O, Huang Y-C, Shoemaker D, et al. A Y-like social chromosome causes alternative colony organization in fire ants. Nature. 2013;493(7434):664-8. doi: https://doi.org/10.1038/nature11832

- Line 363
- citation 43 should actually be 62
- citations 60 & 61 are redundant - cite 43 instead for one of them.

Response: We have corrected these.

- How strong are the gene expression changes (e.g., L356-9 - an 80% change is very different from a 1% change) (the power to detect change may be particularly high for these highly expressed genes - we know this is the case for gp-9s).

Response: Thank you for the comments. *Gp-9* has shown ~85% down-regulation in ds-NPA group compared to control ds-eGFP group, and *KAZALD1* has shown ~1.89 fold up-regulation in ds-NPA group compared to control ds-eGFP group. The exact expression levels of all the DEGs of all samples were included in the supplementary Data.

References

1. Mathiron AG, Dixneuf C, Mondy N, Lécureuil C, Earley RL, Goubault MJH, et al. Ecdysteroids affect female reproductive status and outcome of contest over hosts in the parasitoid wasp Eupelmus vuilleti. Horm Behav. 2020;125:104819. doi: https://doi.org/10.1016/j.yhbeh.2020.104819.
2. Ganter G, Desilets J, Davis-Knowlton J, Panaitiu A, Sweezy M, Sungail J, et al. Drosophila female precopulatory behavior is modulated by ecdysteroids. J Insect Physiol. 2012;58(3):413-9. doi: https://doi.org/10.1016/j.jinsphys.2012.01.004.
3. Gospocic J, Glastad KM, Sheng L, Shields EJ, Berger SL, Bonasio RJC. Kr-h1 maintains distinct caste-specific neurotranscriptomes in response to socially regulated hormones. Cell. 2021;184(23):5807-23. e14. doi: https://doi.org/10.1016/j.cell.2021.10.006.
4. He Q, Zhang Y. Kr-h1, a cornerstone gene in insect life history. Front Physiol. 2022;13:905441. doi: https://doi.org/10.3389/fphys.2022.905441.
5. Inoue S. Neural basis for estrous cycle-dependent control of female behaviors. J Neurosci Res. 2022;176:1-8. doi: https://doi.org/10.1016/j.neures.2021.07.001.
6. West-Eberhard, Jane M. Flexible strategy and social evolution. In: Ito Y, Brown JL, Kikkawa J, editors. Animal societies Theories facts: Japan Scientific Societies Press.; 1987.
7. Jane M, Eberhard W. Wasp societies as microcosms for the study of development and evolution. In: Turillazzi S, West-Eberhard MJ, editors. Natural history evolution of paper-wasps. 400: Oxford University Press Oxford; 1996. p. 290-317.
8. Amdam GV, Norberg K, Fondrk MK, Page Jr REJPotNAoS. Reproductive ground plan may mediate colony-level selection effects on individual foraging behavior in honey bees. Proc Natl Acad Sci USA. 2004;101(31):11350-5. doi: https://doi.org/10.1073/pnas.0403073101.
9. Amdam GV, Csondes A, Fondrk MK, Page Jr REJN. Complex social behaviour derived from maternal reproductive traits. Nature. 2006;439(7072):76-8. doi: https://doi.org/10.1038/nature04340.
10. Gospocic J, Shields EJ, Glastad KM, Lin Y, Penick CA, Yan H, et al. The neuropeptide corazonin controls social behavior and caste identity in ants. Cell. 2017;170(4):748-59. e12. doi: https://doi.org/10.1016/j.cell.2017.07.014.
11. Scharf I, Stoldt M, Libbrecht R, Höpfner AL, Jongepier E, Kever M, et al. Social isolation causes downregulation of immune and stress response genes and behavioural changes in a social insect. Mol Ecol. 2021;30(10):2378-89. doi: https://doi.org/10.1111/mec.15902.
12. Koto A, Tamura M, Wong PS, Aburatani S, Privman E, Stoffel C, et al. Social isolation shortens lifespan through oxidative stress in ants. Nat Commun. 2023;14(1):5493. doi: https://doi.org/10.1038/s41467-023-41140-w.
13. Wang ZY, McKenzie-Smith GC, Liu W, Cho HJ, Pereira T, Dhanerawala Z, et al. Isolation disrupts social interactions and destabilizes brain development in bumblebees. Curr Biol. 2022;32(12):2754-64. e5. doi: https://doi.org/10.1016/j.cub.2022.04.066.
14. Nagel M, Qiu B, Brandenborg LE, Larsen RS, Ning D, Boomsma JJ, et al. The gene expression network regulating queen brain remodeling after insemination and its parallel use in ants with reproductive workers. Sci Adv. 2020;6(38):eaaz5772. doi: https://www.science.org/doi/10.1126/sciadv.aaz5772.
15. Heinze J, Tsuji K. Ant reproductive strategies. Res Popul Ecol. 1995;37(2):135-49. doi: https://doi.org/10.1007/BF02515814.
16. Lattke JE. A Taxonomic Revision and Phylogenetic Analysis of the Ant Genus Gnamptogenys Roger in Southeast Asia and Australasia (Hymenoptera: Formicidae: Ponerinae): Formicidae: Ponerinae. Oakland, CA: Univ of California Press; 2004.
17. Badisco L, Claeys I, Van Hiel M, Clynen E, Huybrechts J, Vandersmissen T, et al. Purification and characterization of an insulin-related peptide in the desert locust, Schistocerca gregaria: immunolocalization, cDNA cloning, transcript profiling and interaction with neuroparsin. J Mol Endocrinol. 2008;40(3):137-50. doi: https://doi.org/10.1677/JME-07-0161.

18. Veenstra JAJIb, biology m. What the loss of the hormone neuroparsin in the melanogaster subgroup of Drosophila can tell us about its function. Insect Biochem Mol Biol. 2010;40(4):354-61. doi: https://doi.org/10.1016/j.ibmb.2010.03.001.
