# Supplementary material for: An evolutionarily conserved pathway mediated by neuroparsin-A regulates reproductive plasticity in ants
Source: PLoS Biol. 2024 Aug 12;22(8):e3002763. doi: 10.1371/journal.pbio.3002763 (PMC11398701; doi:10.1371/journal.pbio.3002763)
Supplement: Attachment — Submitted filename: Response to Reviewers.docx [file pbio.3002763.s022.docx]

Please attend to the following:

a) Please address the remaining comments from the reviewers.
Response: We have addressed the comments from the reviewers in a point-by-point manner.

b) Please make all underlying custom code and data available, according to our data policy (see link further down).
Response: We have made all custom codes and data available.

c) The Academic Editor noted some similarity between the phylogeny in your Fig 1B and that in Fig 1 of ref 17 (Chandra et al). It looks like it has been re-drawn, so there's no copyright issue, but if ref 17 was indeed the source for the phylogeny then it would be good to cite this paper in the legend.
Response: Thank you for bringing to our attention the similarity between Figure 1B in our manuscript and Figure 1 from reference 17 by Chandra et al [1]. We appreciate the opportunity to clarify the source and ensure proper attribution. We acknowledge that the phylogeny depicted in Figure 1B was redrawn independently from reference 17 by Chandra et al [1]. Subsequently, we revisited the original source of the phylogeny and identified it as Borowiec et al [2]. In light of this, we have updated the legend for Figure 1B to include a citation to Borowiec et al, which provides a comprehensive analysis of ant subfamily phylogeny[2]. The study utilized data from 11 nuclear loci across 110 ant species, offering valuable insights into the internal phylogeny of ants, including discussions on the challenges and uncertainties inherent in such analyses. The revised citation in the legend reads as follows:

Borowiec ML, Rabeling C, Brady SG, Fisher BL, Schultz TR, Ward PS. Compositional heterogeneity and outgroup choice influence the internal phylogeny of the ants. Mol Phylogenet Evol. 2019;134:111-21. doi: <https://doi.org/10.1016/j.ympev.2019.01.024>..

We believe this update appropriately acknowledges the original source and enhances the transparency of our work.
------------------------------------

REVIEWERS' COMMENTS:

Reviewer #1:
[identifies himself as Romain Libbrecht]

In the revision of their manuscript, the authors have successfully addressed most of the concerns that I had expressed in my original review. In particular, they have responded to my skepticism related to the behavioral aspects of the study by toning down their interpretation of the behavioral experiments - even removing the behavioral data that I deemed not robust enough because of the experimental design and the method of data collection (see my original review). They have also used a criticism that I made on the possible link between reproduction and behavior - which they had already mentioned in the original manuscript - to reshape their introduction around a possible role of the brain-ovary axis. Then, they have added several ant species to their dataset, which strengthened their comparative analyses. Finally, they improved the writing of the manuscript (note that I still found a small typo L158 and 160: "biori" should be "biroi") and provided the missing details in how they report their statistical analyses.

Overall, I think that these changes greatly improved the manuscript. I thank the authors for the efforts that they have produced in order to address my concerns. I originally recommended the manuscript to be rejected, but I must admit that I was wrong to do so and I am happy that the editors gave the authors the opportunity to resubmit. I find the revised manuscript to be very convincing and, as stated in my original review, I remain very impressed by the fantastic molecular, functional work that is being reported in this study, which I now recommend for publication in Plos Biology.

Romain Libbrecht

Response: We appreciate the reviewer’s positive comments on our study. We have corrected the spelling of *Ooceraea biroi*.


Reviewer #3:

I reviewed the previous version of the manuscript. I have now carefully read the other reviews, the author responses, and the revised manuscript. The study topic is very interesting and the results are relatively straightforward and potentially of broad interest. The revised manuscript is improved by some of the additional studies that were done, and some of the changes to the text. However, I find a number of major issues with the revised manuscript, some of which I did not notice in the original version:

1. The behavioral analyses are flawed.
I agree with Reviewer 1 and Reviewer 4 that the behavioral studies are fundamentally flawed. I did not notice these issues before. As reviewer 4 points out, the statistical analysis needs to explicitly account for the grouping variable by including it as a random factor. The authors responded "Following the reviewer's suggestion, in this revision, we didn't combine the three experiments, instead we presented the results from just one experiment…". This is not an adequate response to the Reviewer 4's comment. As far as I can tell from the Methods, the H. saltator behavioral study used 7 replicate colonies (L313; even though the Methods L681-L688 do not explain replicate colonies, nor L572). In contrast, the behavioral studies in *M. pharaonis* (L244-248 and L666) use 7-9 individual treated queens (?) placed together in one petri dish and a similar number of untreated queens in a second petri dish. Any random environmental factors that affected one petri dish and not the other would be completely confounded with the treatment effect. This is not a suitable study design. Replicate groups (petri dishes) need to be analyzed and group needs to be explicitly included in the statistical model.
Response: Thanks for the reviewer’s comments. We recognized the inadequacies in our descriptions of the material/methodological information and the flawed statistical analysis of the behavioral data. We supplemented the detailed colony replication and methodological information in methods (L699-704). The *Monomorium pharaonis* behavioral experiments were performed in a temperature and humidity-controlled room. The experimental and control groups were put in petri dishes that were immediately close to each other (<20cm), and their behaviors were video-taped during the same period of time, therefore, the impacts of different environmental factors were minimized. We re-analyzed all behavioral data of *M. pharaonis* ants, including locomotion distance, foraging frequency and foraging duration, with a statistical model accounting for the grouping variables. Statistical analyses were performed in an R (v4.1.2) environment. We considered the foraging behaviors in a given time following compound Poisson processes, where behaviors came with associated variables such as duration or walking distance. We employed the R package “cplm” of which the information of replicate groups in our data can be handled to fit a null model (mod0 = cpglm(Value~Replicate)) and an alternative model (mod1 = cpglm(Value~Replicate+Treatment)) [3]. The alternative and the null model were then compared using the waldtest function of the R package “lmtest” and the *P*-value was calculated [4].

We updated the results in Figure3, and modified the relevant main text (line 773-781 in the revised manuscript). All the summarized and raw behavioral data as well as statistical test results are uploaded as supplementary materials and metadata.

2. Methodological details, raw data, and scripts are missing.
In general, I often could not tell exactly what the authors did or how exactly they analyzed their results: as far as I can tell, no scripts or raw data are included, either for the behavioral data or the gene expression data. No mention is made of RNA seq reads being deposited.
Response: We have added the statistical analysis for each experimental treatment in Materials and Methods section, which displayed all the summarized data and statistical methods we used. The raw data for each ovarian oocyte measurement was uploaded in Meta Data. The locomotion raw data consists of coordinates of each individual per frame. We have uploaded all coordinate data of each ant individual for 10, 30 and 60 min in supplementary txt files. For foraging and hunting behavior, we recorded the start and end time point of each foraging, biting and tube wall touching behavior, the raw data was uploaded in Meta Data. We have deposited raw RNA-seq data as “The raw RNA-seq data of brains and ovaries generated in this study” in the CNGB Nucleotide Sequence Archive (CNSA) with accession number CNP0004568. (L695-697)

3. Writing is inconsistent and difficult to follow in places (especially the Abstract, Introduction, Results, and Methods).
I had a hard time following the arguments of the Abstract and Introduction (see more details below). In contrast, I found the Discussion to be generally very well written and clear. Overall, the manuscript is inconsistent with the presentation, which in the end greatly detracts from the interesting results and relatively simple and powerful study design (except for the behavior portion, see above).
Response: We apologize for our inconsistent writing that made the logic flow hard to follow. We have carefully polished our writing in the Abstract, Introduction and Results sections (as detailed below in our response to the reviewer’s minor comments). And we have added more details in the Materials and Methods section to make clear of our experimental manipulations.

Additional comments and suggestions:

L40-41 The first sentence of the abstract is stated as if it is a fact, but it's actually a hypothesis.
Response: We have toned down the argument of this sentence.

"Gyne" is a social insect specific word and should probably be avoided, at least in the Abstract.
Response: Thanks for suggestion. We have replaced “gyne” to “virgin queen (gyne)” in the Abstract, please referring to line 46-47 in the revised manuscript.

L44 (and L423) what does "remarkable gene regulatory network remodeling" mean? Does this simply refer to transcriptomic differences? I'm used to "remodeling" used with respect to GRNs in terms of their evolution, like this paper: https://www.pnas.org/doi/abs/10.1073/pnas.1305457110?doi=10.1073%2Fpnas.1305457110 . "re-wiring" is used in an evolutionary sense in the Discussion, e.g., L544-560, which fits with my understanding of general usage, but the usage of "remodeling" is confusing in my opinion.
Response: Thank you for this suggestion. We have realized the description of the “re-modeling” of the GRN is not appropriate in the context of phenotypic plasticity. We have changed the description of gene regulatory network “remodeling” to gene regulatory network “shift”.

L49 "..mainly targeted on…" is confusing wording. Do you mean that NPA mainly affected..? You showed that injection of NPA peptide affected 5 genes in the brain and 1051 genes in ovaries, so I'm further confused by what "mainly targeted on.." means.
Responds: Sorry for the confusion. We have changed the description of “..is mainly targeted on…” to “..mainly affected..”. In our ovary transcriptomics dataset, we have only identified 20 DEGs that were shared between both NPA-dsRNA and NPA-peptide treatments. Among the 20 shared DEGs, 10 of them were up-regulated in dsRNA treatment while down-regulated in peptide treatment, while other 10 DEGs displayed the opposite variation patterns (S4 Fig). We specially highlighted a few genes due to the following reasons: 1) these genes are among the 20 shared DEGs (differentially expressed in both dsRNA and peptide treatments); 2) they have high log2 Fold change values (Fig 5A); 3) their expressions in the ovary were validated by HCR RNA-FISH (Figure5C); and 4) one of the genes (*JHBP*) was experimentally validated to regulate reproduction (Figure5B).

Sometimes "adjusted P < 0.05" is reported and something "adjusted P < 0.10" is reported. Why? The Methods mentions that that differential expression was analyzed with DESeq2. What exact p adjustment was used? These details need to be specified and relevant scripts used for data analysis need to be included so that reviewers and readers can tell exactly what you analyses you did. Similarly, for GO enrichment with clusterProfiler, please specify what analyses you did, include scripts detailed exactly what you did, and explain how you corrected for multiple comparisons.
Response: Sorry for the missing descriptions of the methodological details. For the transcriptomics analysis, "adjusted *P*< 0.10" was reported for DEGs that are expressed in the brain. It is because the DEGs identified in the brain were very few and the expressions of the DEGs were highly dispersed. We apologize for the inconsistency in our presentation and we have unified to report the adjusted *P* <0.05 in the revised study. We firstly used the Wald test to get the p-value for each gene. Then, the *P*-values are corrected for multiple testing using the default Benjamini and Hochberg method by DESeq2 [5].

For GO enrichment analysis with clusterProfiler (4.2.2), a one-sided version of Fisher’s exact test is performed by enrichGO, the *P*-values are calculated by hypergeometric distribution [6]. We used multiple test correction to correct the *P*-values and used the Benjamini and Hochberg method for correction. We have added differentially expressed genes GO enrichment result in S4 Fig, and the detail of GO enrichment analysis in Methods as “For the GO enrichments of peptide-injected ovarian differentially expressed genes, we used clusterProfiler 4.2.2 with multiple test correction to correct the *P*-value, and choose Benjamini and Hochberg correction method.” (L690-693). The scripts used for DEG and GO enrichment data analysis are included in the Supplementary Material.

L664-L667 refers to "figure generation codes" and notebooks, etc., but as far as I can tell, none of the relevant scripts are actually included, so that it would be impossible for anyone to try to replicate the analyses used in this study.
Response: We have appended the figure generation codes in supplementary material. The details of locomotion behavior analysis are rewritten for readers to understand and replicate the analyses. The rephrased paragraph is as following: “We used the following protocol to analyze locomotion behavior: i) installing Anaconda on 64-Bit Windows, open Anaconda and using installer program, pip, to install opencv-python, numpy, pandas, matplotlib, seaborn and ast, then install trajectory analysis package, trackerao; ii) using Corel Video studio 2021 to accelerate a video to quadruple speed; iii) trajectory tracking was conducted in Adobe After Effects CC 2019 built-in tracker, choose track motion for individual tracking, then edit track target as a new object and link a layer. Each individual and pseudo-nest were represented by color square (Lime, R, G, B=0, 255, 0; Navy, R, G, B=0, 0, 128; Magenta, R, G, B=255, 0, 255), respectively. Since 7-9 ants were contained in one petri-dish, color square setting was performed 3 to 4 times for different ants in the video. Nest as an anchor point for ants in the petri-dish, and 2 to 3 individuals were tracked simultaneously. The tracking video was exported, displaying three colored squares with white background by Adobe Media Encoder CC 2019. All exported files were transformed from MP4 to AVI format in Format Factory; iv) open Anaconda Powershell Prompt entering video path and then typing jupyter notebook for remote connection. Establish a new python3 notebook on the web page, entering codes for starting, then enter processed video path and name (S1 Text). Input squares number and pitch up color squares in sequence in the new ROI frame. The information of pseudo-nest and ant motion coordinates were saved in the same file with processed video after the squares tracking finished; v) establish a new python3 notebook for figure generation and coordinates statistics. Entering start code, then paste ant coordinates from step iv) generated text file, individually (S1 Text). Entering figure generation codes to set up appropriate parameters for dots per inch (dip), figsize, Vmax, min-distance and distance, then operate codes (S1 Text).” (L709-730)

L51-53 I don't understand this sentence. "Co-opted' suggests evolutionarily co-opted, but co-opted from what? What "distantly related species"? To make this sense, I think you would have to explain the function in other insects and then make inferences about the ancestral state.
Response: Sorry for the inappropriate use of “co-opted” out of an evolutionary inference context. We have rewritten this sentence to “Furthermore, we show that NPA was employed into the brain-ovary axis in regulating the worker reproductive changes in other distantly related species, such as *Harpegnathos venator* ants” (L51-53)

L57-59 this is only true for ant species that have colonies initiated by a single queen.
Response: Thanks for the comment. We have rewritten this sentence as “The lifecycle for the majority of ant species begins from insemination of a virgin queen (gyne).” (L57-58)

L96-99 this wording "suggesting the co-option of the same ..GRN..across ant species" suggests multiple independent co-option events. But it seems more likely that this is ancestral to all extant ants.
Response: Thank you for the comment. We rephrased the sentence to “…suggesting that the same gene regulatory network (GRN) was retained to regulate different forms of reproductive role differentiation across ant species.” (L93-98)

L98-L100 "NPA …earliest …suppressed in queen long after insemination".

Please clarify what "earliest" means and also what "long after insemination" means. These are critical introductory points, and these statements are repeated later in the ms, but they are currently unclear.
Response: We have rephrased the sentence to “Among these, *Neuroparsin A* (*NPA*) is one of the top ranked and earliest responsive DEGs for gyne/queen transition (suppressed in five day-old queen who is inseminated for two days), and its expression is suppressed in queen long after insemination (suppressed in 30 day-old queen who is inseminated for 27 days)”. (L98-101)

L108 what does "anti-juvenile hormone" mean?
Response: Sorry for the inappropriate wording, what we mean here is “antagonizing juvenile hormone”, we changed the text accordingly. (L109-111)

Throughout, when referring to "queens", e.g., L138 "...always down-regulated in queens compared to gynes", please clarify: do you mean egg-laying queens, or reproductively mature queens, or simply mated queens?
Response: Most of the queens were collected from the field, and were dissected shortly after they arrived at the laboratory, therefore, we cannot distinguish clearly whether the queens were actively egg-laying or reproductively mature or just mated. Yet, the queens that we used to detect brain NPA expression levels were inseminated (the spermatheca were full), and had yolky oocytes, some ovaries contain yellow bodies.

Fig 1. The estimated 95% CI should be shown instead of 1 SEM.
Response: Thank you for the suggestion. We have updated the plots in Figure 1, showing mean and estimated 95% CI.

L182: What does "a very low level of spontaneous ovary activity" mean? How did you measure it?
Responds: Sorry for the vagueness of the expression. What we mean here is that “a very low level of vitellogenesis activity”, we modified the text accordingly. (L183-184)

L188-189 what does "stagnant ovary activity" mean?
Response: Sorry for the vagueness of the expression. We have rephrased the sentence to “indicating that high expression of *NPA* inhibits reproductive activation in gynes…”. (L187-192)

L338-342 this was pharaoh ant mated queens or unmated gynes or? Please explain the source of the 5-12 brains and ovaries. Were they all from the same colony or? These details are important for understanding if the correct statistical analysis was used.
Response: We apologize for the missing methodological details in the text. Since it was too hard to collect enough simultaneously eclosed gynes from a single colony, we collected the gynes that are eclosed on the same day from multiple colonies, pooled them together, then randomly separated the gynes into two groups.

For transcriptomics analysis after NPA knockdown, we injected one group of gynes with *ds-NPA* on day 4, day 6 and day 8, and dissected their brains/ovaries on day 9 for transcriptomics analysis. While we injected the other group of gynes with *ds-eGFP* following the same protocol with a comparable amount of dsRNA, and dissected their brains/ovaries on day 9 for transcriptomics analysis.

For transcriptomics analysis after peptide treatment, we allowed the gynes to mate with males on day 3 after eclosion. On day 4, we removed the males, then injected one group of queens with NPA peptide on day 5 and day 7, and dissected their brains/ovaries on day 9 for transcriptomics analysis. While we injected the other group of queens with BSA following the same protocol with a comparable amount of proteins, and dissected their brains/ovaries on day 9 for transcriptomics analysis.

Since we used a single brain/ovary for transcriptomics analysis, some RNA samples were of bad qualities and could not be used for subsequent library construction, thus, resulting in a variation of sample numbers from 5-12.

We have added the methodological details in the material and methods section (L666-678), and we also appended an illustrating diagram to Supplementary Figure S3.

L377-378 why is it relevant that the yellow/MRJP family "contain an array of members in honeybee"?
Response: We realized that the multiplication of yellow/MRJP family members in honeybee has no explicit relevance to their reproductive regulatory function that we intend to emphasize in the text. We have rephrased the sentence into “Yellow-g2 and MRJP belong to yellow/MRJP family which play crucial roles for reproductive maturation in honeybee”. (L386-387)

L393 "..conserved and crucial function"... specify that by "conserved" you mean conserved across the ant species you studied, and putatively across all ants.
Response: Based on the results of the study, we would suggest that the functional role of NPA in reproductive role differentiation is putatively conserved across all ants. We have provided the following evidences: 1) NPA expression is consistently found to be suppressed in reproductively activated females across nine different species from five subfamilies; 2)NPA is specifically expressed in Medial Neural Secretory Cells (MNSC) across three reproductive models represented by ants from three distantly related subfamilies; 3) the function of NPA in regulating reproduction and foraging behaviors is validated in *Monomorium pharaonis* (subfamily: Myrmicinae) and *Harpegnathos venator* (subfamily: Ponerinae). The species we studied are broadly scattered in ant phylogenetic tree and covering major evolutionary branches, some of which diverged by more than 100 million years [7]. However, we do acknowledge that the number of species we studied was limited compared to the huge diversity of ants. Therefore, we would make a low-key, yet specific statement by rephrasing our statement to: “ we delineate a conserved and crucial function of the brain peptide NPA …, putatively across ant phylogenetic tree”. (L402-406)

__________________________________________________
In compliance with data protection regulations, you may request that we remove your personal registration details at any time. (Use the following URL: https://www.editorialmanager.com/pbiology/login.asp?a=r). Please contact the publication office if you have any questions.

References

1. Chandra V, Fetter-Pruneda I, Oxley PR, Ritger AL, McKenzie SK, Libbrecht R, et al. Social regulation of insulin signaling and the evolution of eusociality in ants. Science. 2018;361(6400):398-402. doi: <https://www.science.org/doi/10.1126/science.aar5723>.

2. Borowiec ML, Rabeling C, Brady SG, Fisher BL, Schultz TR, Ward PS. Compositional heterogeneity and outgroup choice influence the internal phylogeny of the ants. Mol Phylogenet Evol. 2019;134:111-21. doi: <https://doi.org/10.1016/j.ympev.2019.01.024>.

3. Zhang Y. Likelihood-based and Bayesian methods for Tweedie compound Poisson linear mixed models. Stat Comput. 2013;23:743-57. doi: <https://doi.org/10.1007/s11222-012-9343-7>.

4. Zeileis A, Hothorn T. Diagnostic checking in regression relationships. R News. 2002;2(3):7-10.

5. Love MI, Huber W, Anders S. Moderated estimation of fold change and dispersion for RNA-seq data with DESeq2. Genome Biol. 2014;15(12):1-21. doi: <https://doi.org/10.1186/s13059-014-0550-8>.

6. Boyle EI, Weng S, Gollub J, Jin H, Botstein D, Cherry JM, et al. GO:: TermFinder—open source software for accessing Gene Ontology information and finding significantly enriched Gene Ontology terms associated with a list of genes. Bioinformatics. 2004;20(18):3710-5. doi: <https://doi.org/10.1093/bioinformatics/bth456>.

7. Moreau CS, Bell CD, Vila R, Archibald SB, Pierce NE. Phylogeny of the ants: diversification in the age of angiosperms. Science. 2006;312(5770):101-4. doi: 10.1126/science.112489.
