# Supplementary material for: An evolutionarily conserved pathway mediated by neuroparsin-A regulates reproductive plasticity in ants
Source: PLoS Biol. 2024 Aug 12;22(8):e3002763. doi: 10.1371/journal.pbio.3002763 (PMC11398701; doi:10.1371/journal.pbio.3002763)
Supplement: Attachment — Submitted filename: responds to reviewers.docx [file pbio.3002763.s023.docx]

Reviewer #1:

[identifies himself as Romain Libbrecht]

This is the second revision of this manuscript and my assessment remains similar to the last version. I am very impressed by the fantastic molecular, functional work that is being reported and I am very convinced by the data supporting a role of NPA in ant queen reproduction. However, I am still concerned that the behavioral work is not on par with the molecular work, in particular the grouping of queens together in the same container for behavioral analyses and the interdependency between data points that this experimental design produces. Maybe the authors could consider downplaying the behavioral results even more (currently it is still emphasized in the title, abstract, etc).

Response: Thanks for the reviewer’s comments. We have toned down the behavioral findings throughout our revised manuscript. We have changed the title to "An evolutionarily conserved pathway mediated by neuroparsin-A regulates reproductive plasticity in ants". We have deleted the description of behavioral results in abstract by rephrasing the sentence “Knock-down of *NPA* in virgin queen (gyne) enhances ovary activity and inhibits activity for locomotion and foraging behavior. Conversely, injection of NPA peptide in fertilized queen suppresses ovary activity and causes higher locomotion” into “Knock-down of *NPA* in unmated queen enhances ovary activity, whereas injection of NPA peptide in fertilized queen suppresses ovary activity” (Line 47-48). In discussion, we have rephrased the sentence “In this study, we validated the function of NPA in regulating reproductive physiology and behavior for differentiated gyne/queen roles in pharaoh ants” into “In this study, we validated the function of NPA in regulating reproductive plasticity for differentiated unmated queens and mated queens in pharaoh ants” (Line 435-436). We also discussed the limitations of our behavioral studies by adding the sentence “Our results also suggest a potential role of NPA in regulating locomotion and foraging behaviors in ants. However, because we grouped multiple gynes/queens together in the same petri-dish for behavioral assays, we cannot exclude the effect of grouping variables that cause interdependency between data points. A more rigorous experimental design is needed to definitively determine the role of NPA in behavioral regulation” (Line 436-441).

COMMENTS FROM THE ACADEMIC EDITOR:

I agree with Reviewer 1. Let's have the authors remove the words "and behavior" from the title and de-emphasize the behavioral importance from the rest of the manuscript.

Response: Thanks for the comment. We changed the title and de-emphasized the behavioral results in our revised manuscript. Please refer to our response to reviewer 1 in previous paragraph.

I noticed a few other things that the authors should address as well.

Lines 402 to 406 read like discussion to me. They should consider rewording this or moving it.

Response: We removed this paragraph and reworded the last sentence in the previous paragraph (Line 403-406).

The authors should de-emphasize the behavioral results and possibly discuss the limitations of their behavioral experiments in the discussion section.

Response: we have de-emphasized the behavioral results and discussed the limitations of our behavioral experiments. Please refer to our response to reviewer 1.

For Figure 1B I still don’t see the citation to the phylogeny in the figure legend. I agree that they should cite Borowiec. They also don’t mention the phylogeny at all in the legend and they don’t say where the data comes from and what the circles mean. I think this should be described better.

Response: we have added the citation of the phylogeny in the legend of Figure 1B. We have described the data that was used to construct the phylogeny and explained the meaning of the circles (Line 177-179).
